# Anti-epileptogenic effect of FC99 and resveratrol

**DOI:** 10.3389/fnins.2023.1223196

**Published:** 2023-08-24

**Authors:** Oded Shor, Roy Rabinowitz, Nir Hersh, Alexey Vanichkin, Felix Benninger

**Affiliations:** ^1^Felsenstein Medical Research Center, Beilinson Hospital, Petach Tikva, Israel; ^2^Faculty of Medicine, Tel Aviv University, Tel Aviv, Israel; ^3^Neurology Unit, Sanz Medical Center - Laniado Hospital, Netanya, Israel; ^4^Department of Neurology, Rabin Medical Center, Petach Tikva, Israel

**Keywords:** TLR3 blockers, epilepsy, epileptogenesis, pilocarpine, anti-seizure medication, neuroinflammation, TLR3

## Abstract

Toll-like receptor 3 (TLR3), plays an important role in the development of epilepsy after brain insults. Previously, TLR3 deficiency in a pilocarpine model of temporal lobe epilepsy (TLE) was shown to reduce mortality, spontaneous recurrent seizures (SRS) and neuroinflammation. We hypothesized that pharmacological inhibition of TLR3 would reduce epileptogenesis following status epilepticus. We show that Resveratrol and FC99, two TLR3 blockers, demonstrate anti-epileptogenic effects in a pilocarpine model of TLE. While both Resveratrol and FC99 were previously shown to benefit in other pathologies, neither of these blockers had been proposed for the treatment of epilepsy. Our results provide substantial evidence to the importance of TLR3 inhibition in the prevention of epilepsy and specifically highlighting FC99 as a promising novel anti-epileptic drug. We anticipate our data to be a starting point for further studies assessing the anti-epileptogenic potential of FC99 and other TLR3 blockers, paving the way for pharmacological interventions that prevent epileptogenesis.

## Introduction

Epilepsy is one of the most prevalent neurological disorders, affecting 60 million people worldwide (Engel et al., 2003; Kokaia, 2011). Despite the development of anti-seizure medications (ASM), up to 35% of the patients are drug refractory, possibly requiring surgical intervention (Engel, 1998; Wiebe et al., 2001a,b; Macrodimitris et al., 2011). In many epilepsy patients, brain damage such as trauma, infections, stroke, status epilepticus (SE) and febrile seizures are associated with seizures during the acute phase and increase the risk for development of epilepsy as a chronic disease (epileptogenesis; Herman, 2002; Temkin, 2009). While current non-surgical treatment approaches are focused on reducing the symptoms (i.e., ASM), there are no available pharmacological interventions that prevent epileptogenesis (Engel, 2019). Cumulative evidence from both animal studies and clinical experience in human patients, emphasize the association of the aforementioned brain insults and neuroinflammation (Lozano et al., 2015). The transformation of microglia from resting to activated state (Kokaia, 2011), and the release of proinflammatory effector proteins (Devinsky et al., 2013), play a key role in driving the neuroinflammatory response. Toll-like receptors (TLRs) are a family of innate immune receptors that are potent proinflammatory drivers (Okun et al., 2011). Due to their proinflammatory nature, TLRs may mediate the neuroinflammatory processes that occur in epilepsy. TLR3 is a double-stranded RNA sensor, localized in endosomal compartments. Its activation by synthetic dsRNA polyinosinic-polycytidylic acid [poly (I: C)] causes a long-lasting increase in seizure susceptibility in rats (Galic et al., 2009). In a previous study we have demonstrated that TLR3 deficient mice have reduced epileptogenesis in a pilocarpine model of temporal lobe epilepsy (TLE) with significant reduction of spontaneous recurrent seizures (SRS; Gross et al., 2017). Here, we sought to identify a pharmacological treatment based on our hypothesis that TLR3 inhibition would improve the clinical outcome to prevent epileptogenesis following brain insult. Resveratrol (RESV; 3,5,4′-tri-hydroxy stilbene) is a type of polyphenol and an antimicrobial substance synthesized *de novo* by plants. A number of beneficial effects of RESV have been proposed, including anti-ischemic, antiviral, antioxidant and anti-inflammatory properties (Baur and Sinclair, 2006). Additional effects by RESV unrelated to TLR3-R might be encountered and thus, the use of small molecules functioning as selective TLR3-R inhibitors may be preferred. In a previous study, FC-99, a novel 1,2-benzenediamine derivative and a TLR3 blocker exhibited an inhibitory effect on the inflammatory response in a mouse model of sepsis (Gong et al., 2014). In this study we sought to assess the potential of RESV and FC99 in preventing epileptogenesis and their effect on frequency and duration of seizures using the pilocarpine model of TLE.

## Methods

### Mice

Animals are used in full compliance with the National Institutes of Health/Institutional Animal Care and Use Committee guidelines. All animal studies were approved by the Animal Care and Use Committee of the Tel Aviv University, Tel Aviv, Israel (Ethic approval # 011116). For the research and control animals, adult male C57BL/6 mice (Harlan, Jerusalem, Israel), 8 weeks old, are used in this study. The establishment of a colony of mice was performed at our institute. All mice are maintained at a mean room temperature of 23°C ± 2°C on a 12-/12-h light/dark cycle. Food and water were provided *ad libitum*.

### Induction of SE with pilocarpine

SE was induced in mice as previously reported (Gross et al., 2017). Briefly, mice were injected subcutaneously (s.c.) with a single convulsant dose of pilocarpine (340 mg/kg, Sigma, Israel) and SE was terminated after 40 min by an intra-peritoneal (i.p.) diazepam injection (4 mg/kg, Teva, Israel). To minimize peripheral muscarinic stimulation, methyl-scopolamine (1 mg/kg, Sigma, Israel) was administered s.c. prior to pilocarpine injection. After a latent period of 1–2 weeks, the SE in this model triggers the process of epileptogenesis leading to chronic epilepsy. The sham group of mice was not injected with pilocarpine but monitored as described below.

### Telemetric electro-encephalogram recordings and seizure classification

Electroencephalogram (EEG) was captured using the small animal telemetry system (Millar Instruments, Houston, TX). Mice were anesthetized (10 mg/kg ketamine, 100 mg/kg xylazine) during the implantation of the transmitter to the right side of the abdominal cavity. Electrodes are then positioned using a stereotactic frame (p.a.: −1.5, m.l.: ±1.5). EEG recording with a sampling rate of 1 kHz was started at day 14 after SE, when mice had recovered from transmitter implantation and stable EEG signals were obtained. Once stable EEG signals were obtained, monitoring was continuously performed for 48 h. EEG recordings were analyzed using the EEGgui matlab toolbox (Sick et al., 2013) for the detection of seizure activity. Spectrogram analyses were accomplished with Matlab software (Mathworks, Natick, MA) using Fast Fourier transformation analysis (FFT). To perform a time-dependent analysis, we used a moving-window approach. EEG signals were divided into non-overlapping segments of 8,192 sampling points each, corresponding to a window length of 8.2 s at the given sampling rate. Prior to FFT calculation, data preprocessing was performed for each window. The duration of increased EEG activity was determined as the time during which band power was increased to 10% of maximal values.

### RESV and FC99

One day following SE induction, mice received six i.p. injections over the course of 2 weeks with either RESV (trans-resveratrol) at 20 mg/kg (Sigma-Aldrich Ltd., Rehovot, Israel), FC-99,100 mg/kg (N1-[(4-methoxy)methyl]-4-methyl-1,2-Benzenediamine; Axonmedcam, Groningen, Netherlands), or DMSO (Dimethyl Sulfoxide, Sigma-Aldrich Ltd., Rehovot, Israel). Final injection solution of DMSO was diluted to include <5% DMSO.

### Statistical tests

The GraphPad Prism v9.4.1 software was used to analyze data. Statistical tests were performed as described in the figure legends and *p* values of ≤0.05 are labeled with a single asterisk (*), in contrast to p values of ≤0.01 (**), ≤0.001 (***), or ≤ 0.0001 (****); where not indicated, p values are non-significant. In all relevant figure panels, values of mean ± SEM are reported, and the exact n value is described in each figure legend. Outliers analysis was performed to exclude such data in each analysis. Sample size: RESV (*N* = 8), FC99 (*N* = 8), DMSO (*N* = 10), and Sham (*N* = 8).

## Results

**Figure 1:**
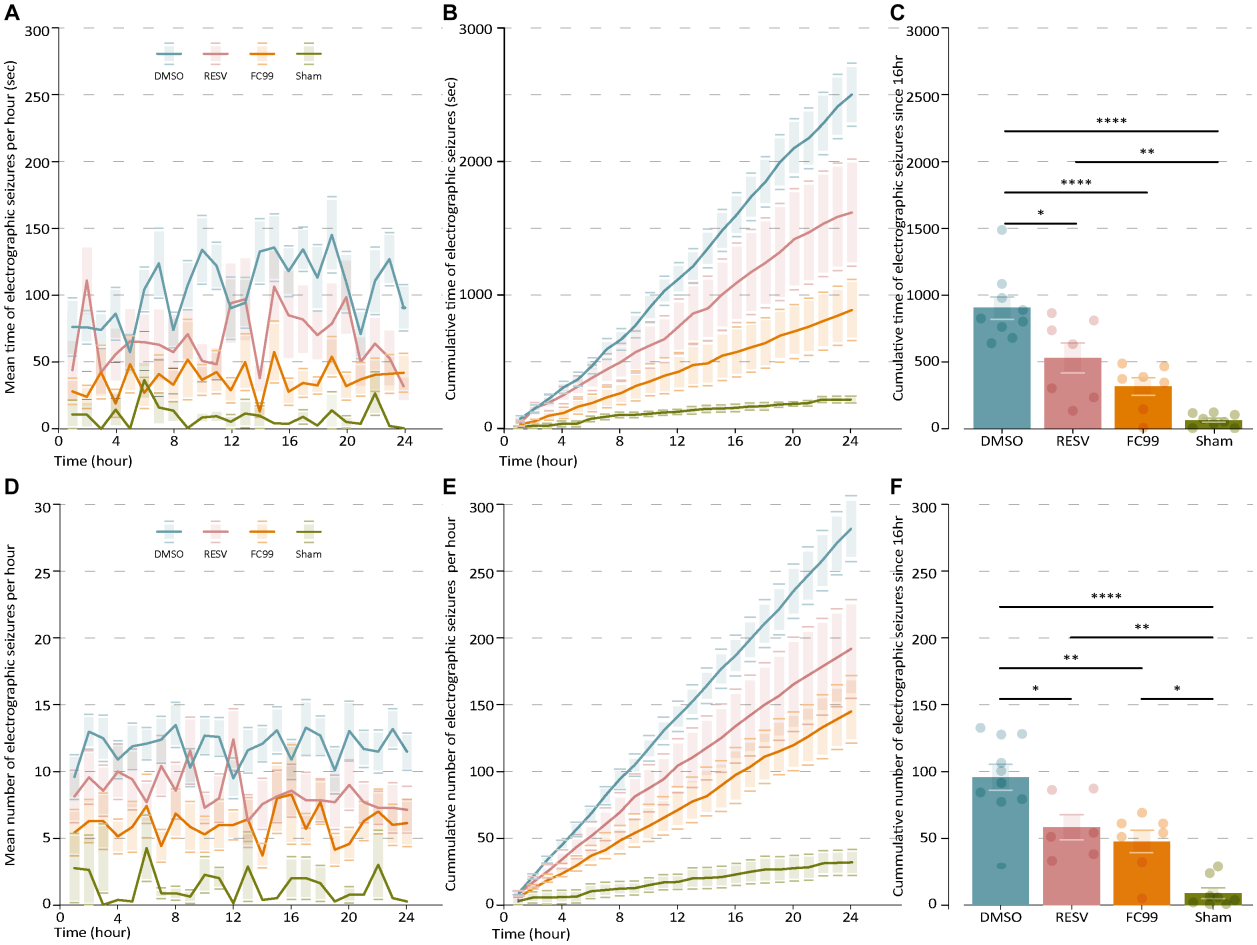
FC99 and RESV reduce the frequency and duration of spontaneous recurrent seizures (SRS). **(A)** Mean duration of electrographic seizures over time. Non-treated mice (DMSO, blue) are compared to RESV (red) or FC99 (orange) treated mice and sham mice (green). **(B)** Cumulative duration of seizures over time. **(C)** Mean cumulative duration of seizures over time. **(D)** Mean number of electrographic seizures over time. **(E)** Cumulative number of seizures over time. **(F)** Mean cumulative number of seizures over time. In the bar plots **(C,F)** non-significant results are not indicated. One-way ANOVA, Multiple comparison correction by Tukey. ɑ = 0.05. Error bars represent the SEM. Raw data and statistical analysis are available (Supplementary material). *p < 0.05; **p < 0.01; ****p < 0.0001.

We hypothesized that early pharmacological intervention aiming to block TLR3 post-SE induction, would lead to improved clinical outcome and may prevent epileptogenesis in this model of TLE. To test this, 1 day post induction of SE by pilocarpine, mice were treated with either RESV or FC99 (both solved in 5% DMSO). A control group was treated with DMSO. Mice were injected every second day (in total of six times) over the course of 12 days. Of the pilocarpine-injected mice, only those that developed clinical SE including whole body tonic–clonic seizures with loss of posture or jumping were subsequently treated and further analyzed (39 mice out of 45, 86.7%). Out of the mice which showed SE, 15 mice succumbed during SE or during the 24 h following SE, leaving 24 mice in the experiment groups. Following the treatment period, on day 12 or 13, mice were implanted with wireless telemetric EEG transmitters coupled to a video recording system. The EEG/Video Telemetry System allowed us to monitor and record multiple animals in parallel for long durations, while obtaining information on their activity and body postures in the cage. EEG recording started 2 days after implantation on day 14 or 15. Using the EEG and the video recording we were able to follow the disease progression and assess the frequency, duration, and power of the spontaneous recurrent seizures (SRSs). By comparing the frequency and mean time of the SRSs, we found significant differences between the experimental groups compared to the vehicle (DMSO-treated) and sham groups (Figure 1). It can be clearly observed that both treatments (RESV and FC99) greatly decreased the frequency and mean time of SRSs, reducing them almost to the level of the sham control group. SRSs occurrences are distributed randomly along the time series measurement. The phenotype of the treated mice remained constant along all times of the measurement (Figures 1A,D). In a cumulative analysis of the SRSs frequency and duration, we identified the window of maximum effect in which the cumulative frequency and duration of the treated mice reached significant differences compared to the vehicle group (Figures 1B,E). By subtracting the final value of total number or time of SRSs in 24 h by the value after 16 h, we obtained a max-score that allows us to determine the effect of each treatment (Figures 1C,F). The max-score for SRSs duration of the vehicle group was 902.33, compared to 528.28 and 314.14 of the RESV and FC99 groups, respectively, and 62.62 for the sham group (DMSO-RESV *p*-val: 0.0102; DMSO-FC99 *p*-val: <0.0001; DMSO-Sham *p*-val: <0.0001).

Similar effects were observed for the frequency of the SRSs. The mean frequency of SRSs (Figure 1D) and cumulative frequency (Figure 1E) analyses demonstrate significant differences between the treatment groups compared to the vehicle group in most time points. We calculated the max-score as described above (Figure 1F) and found significant changes for both treatments compared to the control group (DMSO-RESV *p*-val: 0.258; DMSO-FC99 *p*-val: 0.0019; DMSO-Sham *p*-val: <0.0001). No significant changes were observed between the RESV and FC99 groups (cumulative duration *p*-val: 0.2221; cumulative frequency *p*-val: 0.8522), although significant changes in favor of the FC99 treatment were observed at several time points in both the frequency and duration analyses (Figure 1). Noteworthy, as a result of the FC99 treatment, seizure duration levels were almost as low as in the sham group.

**Figure 2:**
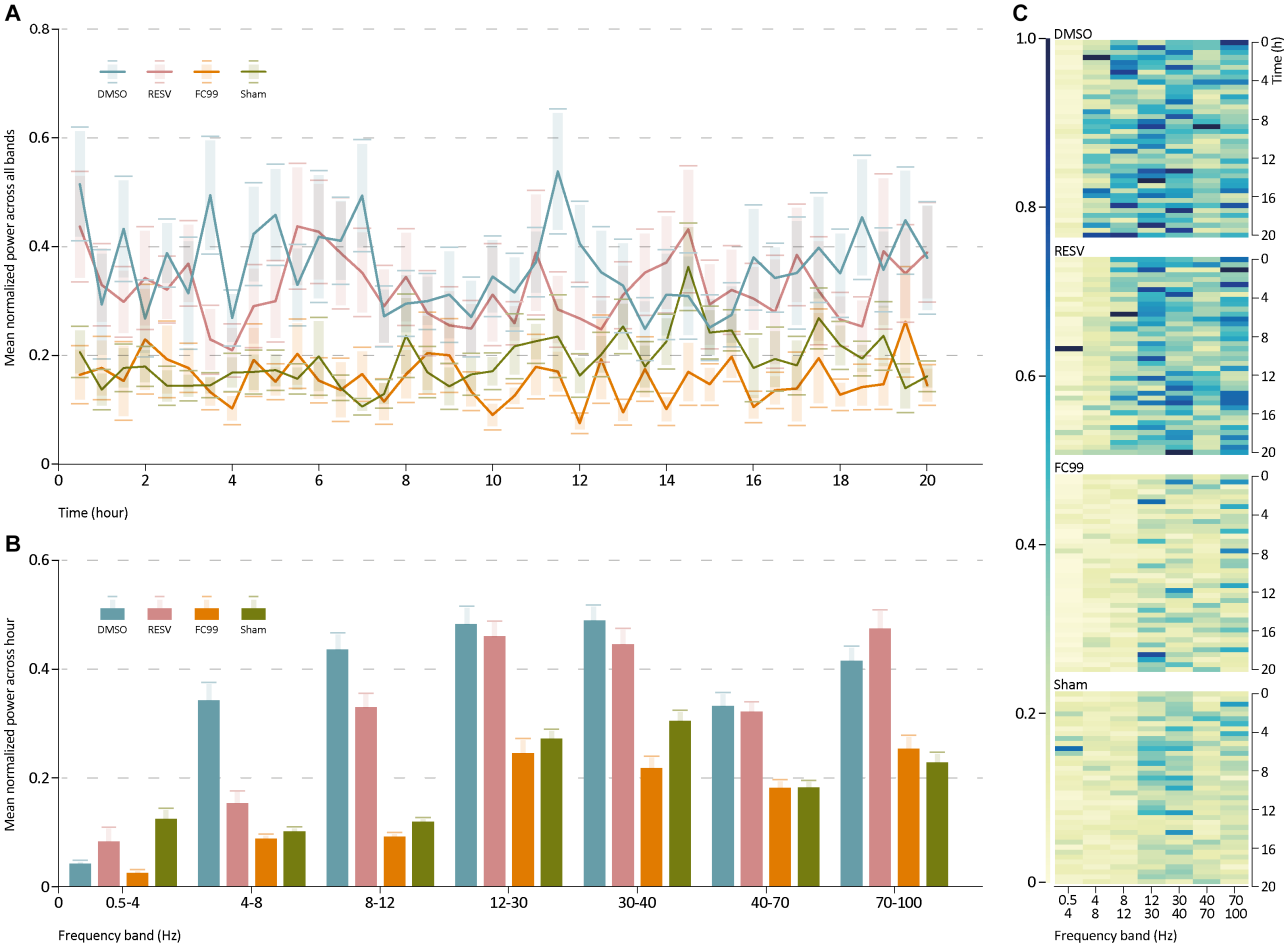
Power spectrum analysis. **(A)** Mean normalized average power across all bands over time. **(B)** Mean normalized average of every group in each frequency band. Statistics reported in Supplementary material. **(C)** Heatmap representation for each group, describing the group’s normalized average spectrogram in each band over time. The rows show the time frames and the columns the frequencies (Hz). Raw data and statistical analysis are available (Supplementary material).

Further, we investigated the distribution of power into frequency components composing the EEG signal of each group (Figure 2). For that purpose, we performed spectral analysis in the seven primary bands (0.5–4 Hz, 4–8 Hz, 8–12 Hz, 12–30 Hz, 30–40 Hz, 40–70 Hz, 70–100 Hz). We used a sliding window of 1 h duration with 30 min overlap. The spectrogram of each group of mice was then averaged across all mice in that group. This analysis was normalized by dividing the average power spectrum of all groups in each band by the maximum power of all groups in this band. The results (Figure 2B) indicate high average normalized power in the DMSO treated mice group in all bands compared to the sham group. Both treatment groups (RESV and FC99) demonstrated significantly lower average normalized power relative to the DMSO group, while the effect was greater in the FC99 group. These results are in consistency with the previous data, describing the dramatic effect of epileptic activity reduction due to FC99.

## Discussion

It is imperative to seek innovative pathways that will hasten novel therapeutic interventions to prevent epilepsy. Early interference in the inflammatory processes, possibly by pharmacological intervention in the TLR pathway, may prevent the events that occur during the latent period after a brain insult (epileptogenesis). In this study we have tested two molecules affecting the TLR3 pathway; RESV inhibits TLR3 while it is suggested that it interacts with a broad spectrum of targets. In contrast, FC99 is a TLR3 specific blocker. Using EEG recordings and seizure classification, we were able to assess the therapeutic effect of these TLR3 blockers to reduce epileptic activity in a pilocarpine model in rats. Confirming our hypothesis, the data in this study demonstrate the anti-epileptogenic effect of FC99 and RESV. These findings also support our previous report on the role of TLR3-mediated neuroinflammation in epileptogenesis. While both treatments resulted in significant improvement (i.e., reduction in epileptic activity), FC99 led to a greater improvement in all measured parameters. This could be due to its specific binding to TLR3 or possibly due to a greater affinity to TLR3 compared with RESV. Future studies on FC99 and RESV kinetics could shed light on this yet to be understood varied effect. Our results demonstrate the clinical potential of RESV and FC99 as an anti-epileptogenic preventative treatment post brain insults. The clinical benefits of these and other TLR3 blockers should be further studied to develop novel therapeutics for epilepsy. It is noteworthy that our study is limited to a specific methodology which produces various measurable parameters (i.e., survival, seizures duration and frequency, spectral power analysis). Future studies addressing the mechanisms underlying the role of TLR3 inhibition in epileptogenesis prevention will broaden our understanding of the processes involved in neuroinflammation-mediated epilepsy and pave the path toward novel and improved anti-epilepsy drugs.

## Data availability statement

The original contributions presented in the study are included in the article/Supplementary material, further inquiries can be directed to the corresponding author.

## Ethics statement

The animal study was approved by Animal Care and Use Committee of the Tel Aviv University, Tel Aviv, Israel. The study was conducted in accordance with the local legislation and institutional requirements.

## Author contributions

FB conceptualized and supervised the study. OS and RR analyzed the data. RR wrote the manuscript. NH and AV conducted the animal procedures. All authors contributed to the article and approved the submitted version.

## Funding

This research was supported by the Israel Science Foundation (grant no. 1010/16).

## Conflict of interest

FB is a consultant for NeuroHelp. None of these companies had any input into the design, execution, analyses or writing of this manuscript.

The remaining authors declare that the research was conducted in the absence of any commercial or financial relationships that could be construed as a potential conflict of interest.

## Publisher’s note

All claims expressed in this article are solely those of the authors and do not necessarily represent those of their affiliated organizations, or those of the publisher, the editors and the reviewers. Any product that may be evaluated in this article, or claim that may be made by its manufacturer, is not guaranteed or endorsed by the publisher.

## Supplementary material

The Supplementary material for this article can be found online at: https://www.frontiersin.org/articles/10.3389/fnins.2023.1223196/full#supplementary-material
